# Knowledge of Mange among Masai Pastoralists in Kenya

**DOI:** 10.1371/journal.pone.0043342

**Published:** 2012-08-17

**Authors:** Francis Gakuya, Jackson Ombui, Jorg Heukelbach, Ndichu Maingi, Gerald Muchemi, William Ogara, Domnic Mijele, Samer Alasaad

**Affiliations:** 1 Department of Veterinary and Capture Services, Kenya Wildlife Service, Nairobi, Kenya; 2 Department of Public Health, Pharmacology and Toxicology, University of Nairobi, Nairobi, Kenya; 3 Department of Community Health, School of Medicine, Federal University of Ceará, Ceará, Brazil; 4 Department of Pathology and Microbiology, University of Nairobi, Nairobi, Kenya; 5 Estación Biológica de Doñana, Consejo Superior de Investigaciones Científicas (CSIC), Sevilla, Spain; 6 Institute of Evolutionary Biology and Environmental Studies (IEU), University of Zürich, Zürich, Switzerland; University of Hong Kong, Hong Kong

## Abstract

**Background:**

Pastoralists in low-income countries usually live in close proximity to their animals and thus represent an important repository of information about livestock disease. Since wild and domestic animals often mix freely whilst grazing, pastoralists are also able to observe first-hand the diseases that are present in wildlife and as such are key informants in disease outbreaks in sylvatic animals. We report here the findings of the first study of the knowledge and role of Masai pastoralists in mange in wildlife and livestock in Masai Mara, Kenya.

**Methodology/Principal Findings:**

In this paper we describe the knowledge of mange accrued by 56 Masai pastoralists in Kenya and how they respond to it in both wildlife and livestock. In total, 52 (93%) pastoralists had a clear idea of the clinical appearance of mange, 13 (23%) understood its aetiology and 37 (66%) knew that mites were the causal agent. Thirty-nine (69%) believed that mange cross-infection between domestic and wild animals occurs, while 48 (85%) had observed mange in domestic animals including sheep (77%), goats (57%), dogs (24%) and cattle (14%). The pastoralists had also observed wild animals infected with mange, above all lions (19%), gazelles (14%), cheetahs (12%) and wildebeests (2%). In 68% of cases Masai pastoralists treat mange infection or apply control measures, most commonly via the topical use of acaricides (29%) and/or the reporting of the outbreak to the veterinary authorities (21%). In the period 2007–2011, Kenya Wildlife Service received 24 warnings of 59 wild animals with mange-like lesions from the Masai Mara pastoralist community. The reported species were cheetah, lion, wild dog, Thomson’s gazelle and wildebeest.

**Conclusion:**

Masai pastoralists have good knowledge of mange epidemiology and treatment. Their observations and the treatments they apply are valuable in the control of this disease in both wild and domestic animals.

## Introduction

Mange is a highly contagious skin disease caused by one or a combination of several species of mites [1]. Mites affect both domestic animals and humans, but also wildlife of zoonotic importance [2], [3]. The most common mite species in wild and domestic animals in Kenya is *Sarcoptes scabiei*. This parasite is a ubiquitous ectoparasite that infects more than 100 species of mammals worldwide [3], [4]. In humans it is known to cause considerable morbidity in a number of different counties [5], [6] and epidemics can be caused by contagion from a single case of scabies in crowded living conditions [7]. Sarcoptic mange may lead to considerable economic losses in domestic animals [8] with repercussions for the animal trade [9]. It also has devastating consequences for wild animals, above all in isolated populations [10], [11], a situation that is worsening due to the limitations of available chemotherapy [12]–[14].

An appropriate disease control program against mites should take into account the entire ecosystem and thus integrate measures targeting both wildlife and livestock [15]. Disease control in domestic animals may be able to interrupt mange transmission to wild animals and vice versa [14], [16].

Recently, attempts have been made to understand mange molecular epidemiology using genetic tools to differentiate between isolates from different hosts and geographical regions [10], [17]–[24]. The epidemiology of mange is still not well understood and seems to differ between animal species and areas of the world [25].

Although mange is well known in the Masai Mara ecosystem [26]–[29], no data is available regarding how much pastoralists know about this disease. As the pastoralists use a number of different methods of controlling disease in their livestock, it is imperative to gather information on how they control mange in this ecosystem. They usually live in close contact with their animals and are an important repository of information about the challenges their animals have to face. Since wild and domestic animals mix freely during grazing, they also have first-hand knowledge of wildlife. Most of the reports of mange-infected wildlife that reach the veterinary department of the Kenya Wildlife Service are received from tour operators and wildlife officers (Veterinary Field Reports). Reports from areas outside the protected areas are brought to the attention of wildlife officers by pastoralists who represent a valuable source of information regarding the presence of mange in wild animals. Thus, the aim of this study was to evaluate the knowledge and practices of Masai pastoralists regarding mange and the repercussions they have on disease management. To date no study has ever evaluated the extent to which local pastoralists’ knowledge and understanding of mange in wildlife and livestock might play a role in mange management and control.

## Methods

### Study Area: Masai Mara National Reserve, Kenya

**Figure 1:**
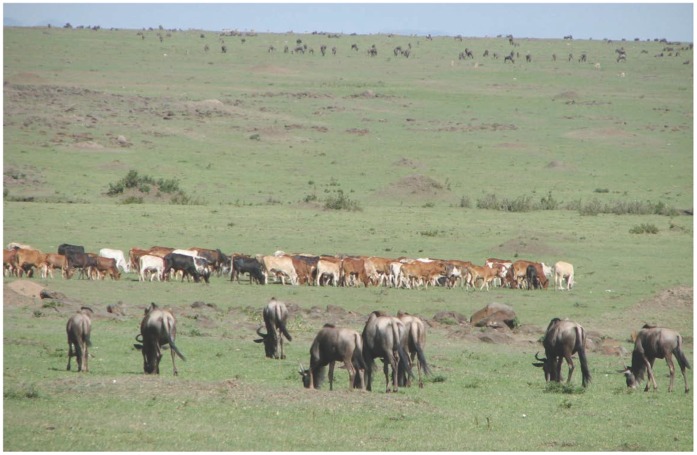
Mixed wildlife and livestock grazing.

This 1510 km^2^ National Reserve is situated in SW Kenya and is effectively the northern continuation of the Serengeti National Park in Tanzania. Rainfall increases along a southeast–northwest gradient and rainy seasons are markedly bimodal. The terrain of the reserve is primarily open grassland with seasonal watercourses. All members of the ‘Big Five’ group of game species (lion, leopard, African elephant, African buffalo and Black Rhinoceros) are present. The millions of wildebeest that dominate Masai Mara migrate northwards in July from the Serengeti plains in search of fresh pasture, before returning southwards in October. This great migration involves some 1,300,000 wildebeest, 500,000 Thomson’s gazelles, 97,000 topi, 18,000 elands, and 200,000 zebras. The migrants are followed along their annual circular route by predators, most notably lions and hyenas. Numerous other antelope species are also found in the National Reserve, including Grant’s gazelle, impalas, topi, elands, duikers, Coke’s hartebeests, zebras and Masai giraffes. The Masai people living around the Masai Mara National Reserve depend on livestock for their livelihoods. Pastoral livestock rearing is the dominant production system in this area, which is characterised by intensive wildlife-livestock-human interaction that includes the sharing of pasture and water (Fig. 1). The livestock species consist mainly of goats, camels, cattle and sheep.

### Study Population and Design

The register of the Kenya Wildlife Service (KWS) lists 150 male householder pastoralists (commonly referred to as Manyattas) aged over 18 years and living within five kilometres of the reserve boundary who essentially form the interface of human-wildlife-livestock interactions. All of the registered households own livestock. From this register, we randomly selected 56 respondents who were invited during visits to their dwellings or via mobile phone to participate in this study. We included only male heads of families as in traditional Masai culture women are not authorised to discuss livestock with visitors or traders without consulting their husbands. The ages of the chosen pastoralists ranged between 18 and 50 years old. Interviews were carried out over a period of one month (June and July 2008) and were conducted by two veterinarians from KWS who are experts on animal disease and proficient in the Masai language. Each interview (conducted in Swahili) lasted approximately 30 minutes. As most of the respondents had either little educational background or were illiterate, questions were read out aloud.

The questionnaire contained (i) structured questions with binary variables and (ii) semi-structured questions with both binary variables (‘yes’ or ‘no’) followed by an open question in which respondents were free to provide open responses (for more details, see supplementary material). The first set of questions aimed to assess pastoralists’ basic knowledge of mange. Respondents were asked if they had ever heard of a disease called mange in any animal, irrespective of whether it was a domestic or wild animal, and if they knew its origin. They were also asked to mention if they had ever heard of parasites known as mites. Another set of questions was used to investigate whether any of the respondent’s animals had ever suffered from mange, whether they were aware of any infected wild animals, and to what extent they were aware of the transmission of mange at the wildlife-livestock interface, above all in light of the fact that livestock and wild animals interact regularly during grazing or when drinking. Finally, participants were asked to mention what control and preventive measures could be used to combat mange and which methods they used (Table S1). Additionally, we documented all warnings of the appearance of mangy wild animals reported by pastoralists from Masai Mara to the Department of Veterinary and Capture Services of Kenya Wildlife Service that occurred between March 2007 and June 2011.

### Capture of Mange-infected Animals and Parasite Identification

Most of the reported animals were captured by chemical immobilization through darting using etorphine hydrochloride (M99® 9.8 mg/ml, Novartis South Africa Pty Ltd, Isando, South Africa) combined with Xylazine hydrochloride (Ilium Xylazil-100 100 mg/ml, Troy Laboratories Pty Ltd, Smithfield, Australia). After sampling and treatment, the animals were revived with diprenorphine hydrochloride (M5050® 12 mg/ml, Novartis South Africa Pty Ltd, Isando, South Africa) and Atipamezole hydrochloride (Antisedan® 5 mg/ml, Pfizer laboratories Pty Ltd, Sandton, South Africa).

Affected areas of the skin were scraped with a scalpel until bleeding to obtain crusts for parasitological examination [30]. Scrapings were placed in universal bottles containing 70% ethanol and transported to the laboratory. All mites were identified on the basis of known morphological criteria [31], [32].

### Mange Treatment

Infected animals were given 1% ivermectin (Kalamectin 1% w/v, Kela NV, St. Lenaartseweg, Belgium), administered sub-cutaneously. Dexamethasone (Glucortin-20® 2 mg/ml, Interchemie, Castenray, Netherlands), an anti-inflammatory and antipruritic drug, was also used. On affected areas of skin, a broad-spectrum oxytetracycline based antibiotic (Alamycin LA® 200 mg/ml, Norbrook Laboratories Ltd, Newry, North Ireland) was applied to prevent bacterial superinfections.

### Ethics

The pastoralists in our study either personally gave their written consent to be included in the survey or, in the case of illiterate pastoralists or those with little educational background, the appropriate document was signed by their families on their behalf. The ethics committee of the Department of Veterinary and Capture Services of the Kenya Wildlife Service (KWS) approved the study and the animal capture protocols. KWS guidelines on Wildlife Veterinary Practice-2006 were followed. All KWS veterinarians follow the Veterinary Surgeons and Veterinary Para-Professionals Act 2011, Laws of Kenya, which regulates veterinary practices in Kenya.

## Results

As defined by the study design, all interviewed pastoralists were male. The mean age was 31.3±8.75 years. Thirty-one (55%) of the respondents were unable to read or to understand the questionnaires and so were assumed to be illiterate. Of the 56 pastoralists interviewed, 52 (92%) had a clear idea of the clinical appearance of mange. However, only 37 (66%) of these pastoralists understood the aetiology of mange (the respondents confirmed that they knew the cause of mange, i.e. that it is caused by small microscopic parasites not visible to the naked eye that could burrow under the skin), while 13 (23%) knew that mites were the causal agent (i.e. they stated that *ilpepedo*, the local name for the mite, is the origin of mange in animals). Thirty-nine (69%) believed that the cross-infection of mange between domestic and wild animals could occur (Table 1). When asked if their domestic animals had ever been affected by mange, 48 (85%) of them answered affirmatively. The animals involved were most commonly sheep and goats (Table 2). Interestingly, the pastoralists had also observed wild animals infected with mange, above all lions, gazelles, and cheetahs (Table 2).

**Table 1:** Pastoralists’ knowledge of mange and treatment and control practices (n = 56).

	No. pastoralists	%
Knowledge of mange	52	93
Knowledge of the aetiology of mange	37	66
Knowledge of mites	13	23
Belief in cross-infection between domestic animals and wildlife	39	70
Seen infected domestic and/or wildlife (for more details see Table 2)	48	86
Practiced treatment or control measures (see text for details)	38	68

**Table 2:** Animal species that pastoralists identified as being affected by mange.

Animal species	N	%
Sheep (*Ovis aries*)	43	78
Goats (*Capra hircus*)	32	58
Dogs (*Canis lupus familiaris*)	14	24
Lions (*Panthera leo*)	11	20
Thomson’s gazelle (*Gazella thomsonii*)	8	15
Cattle (*Bos indicus*)	8	14
Cheetah (*Acinonyx jubatus*)	7	12
Wildebeest (*Connochaetes taurinus*)	2	2
Wild dogs (*Lycaon pictus)*)	1	0.5

**Figure 2:**
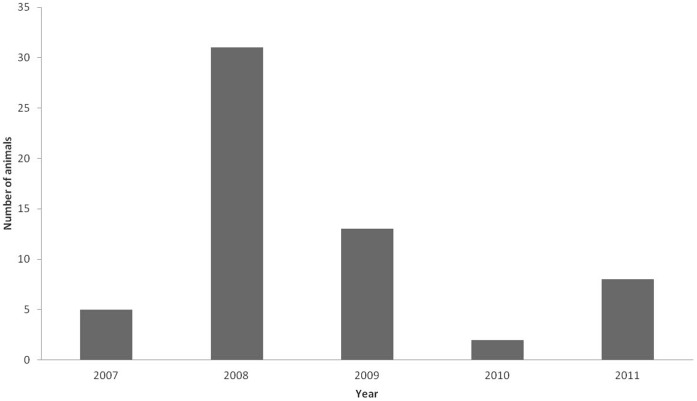
Number of wild animals with mange reported by pastoralists per year (2007–2011).

**Figure 3:**
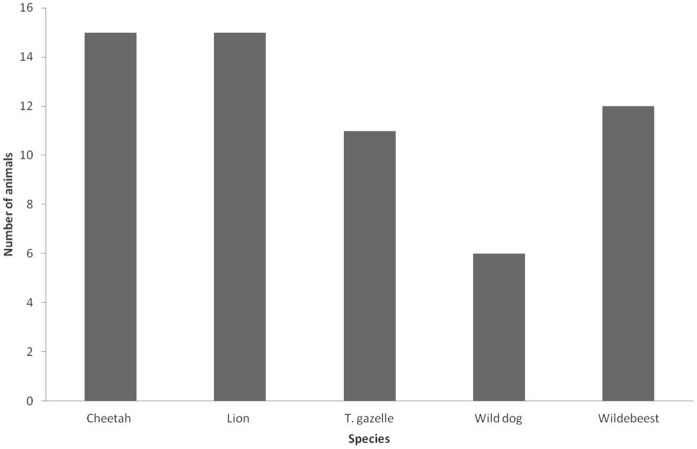
Mange reports for each wild animal species reported by pastoralists (2007–2011).

Between March 2007 and June 2011, the KWS department of Veterinary and Capture Services received 24 alerts about 59 wild animals with mange-like lesions from the Masai Mara pastoralist community, of which 19 were received by phone and five in person from pastoralists. Most reports occurred in 2008 (31/59; 52%), followed by 2009 with 13 reports (22%); there was a lower level of reporting in the other years (5/59; 8%), (2/59; 3%) and (8/59; 13%) in 2007, 2010 and 2011, respectively (Fig. 2). During the five-year period, the following wild species reported to have mange: cheetah, lion, wild dog, Thomson’s gazelle and wildebeest (Figure 3). Most reported cases were of cheetahs or lions (15/59; 25% each), followed by wildebeests (12/59; 20%), Thomson’s gazelles (11/59; 18%) and wild dogs (6/59; 10%).

Most of the pastoralists’ reports came from the Masai Mara National Reserve (68%), Mara Triangle (24%), Olare Orok conservancy (5%) or Ol Choro Oiroua conservancy (3%). Infected animals were captured and treated with ivermectin (Table 3). All the 59 mangy wild animals reported by the Masai people were confirmed as being infected by *Sarcoptes* mite via both the observation of clinical signs and the collection of adult *Sarcoptes scabiei*. Infection in domestic animals was also confirmed by clinical signs and the collection of *Sarcoptes scabiei* from cattle and goats, and *Psoroptes ovis* from sheep.

**Table 3:** Mange alerts reported by Masai people, the date of report, animal data and geographical locality, together with the mode of report and the Kenya Wildlife Service staff who received the alert.

Date	Animalspecies	No. animals, ageclass and sex	Location	Reported to	Mode of reporting
March 2007	Cheetah	3 adult males	Maasai Mara National Reserve	KWS veterinaries	Telephone call
August 2007	Cheetah	1 adult male	Mara Triangle	KWS veterinaries	Telephone call
September 2007	Cheetah	1 adult female	Olare Orok WC	KWS veterinaries	Telephone call
January 2008	T. gazelle	2 adult males	Maasai Mara National Reserve (Talek)	KWS veterinaries	In person
January 2008	T. gazelle	4 Adult males	Maasai Mara National Reserve (Figtree)	KWS veterinaries	In person
January 2008	T. gazelle	4 Adult males	Maasai Mara National Reserve (Figtree)	KWS veterinaries	In person
April 2008	T. gazelle	1 adult male	Maasai Mara National Reserve (Talek)	KWS veterinaries	In person
July 2008	Cheetah	1 adult male	Olare Orok WC	KWS Rangers	Telephone call
August 2008	Cheetah	1 adult male	Olare Orok WC	KWS veterinaries	Telephone call
August 2008	Cheetah	3 adult males	Maasai Mara National Reserve	KWS Rangers	Telephone call
September 2008	Lion	2 cubs (female & male)	Maasai Mara National Reserve	KWS Rangers	Telephone call
October 2008	Wildebeest	12 calves (6 females & 6 males)	Mara Triangle	KWS veterinaries	Telephone call
October 2008	Cheetah	1 Adult female	Mara Triangle	KWS veterinaries	Telephone call
February 2009	Wild dog	1 Adult female	Maasai Mara National Reserve	KWS Rangers	In person
June 2009	Wild dog	5 Adult (2 males & 3 females)	Maasai Mara National Reserve (Figtree)	KWS veterinaries	Telephone call
August 2009	Lion	2 cubs Females	Maasai Mara National Reserve	KWS Rangers	Telephone call
September 2009	Cheetah	1 adult female	Maasai Mara National Reserve	KWS Rangers	Telephone call
December 2009	Lion	4 cubs (2 males & 2 females)	Maasai Mara National Reserve	KWS Rangers	Telephone call
June 2010	Cheetah	1 Adult male	Olchoro-oiroua	KWS veterinaries	Telephone call
August 2010	Cheetah	1 Adult male	Olchoro-oiroua	KWS veterinaries	Telephone call
Februrary 2011	Cheetah	1 adult male	Maasai Mara National Reserve	KWS veterinaries	Telephone call
February 2011	Lion	3 cubs (2 males & 1 female)	Maasai Mara National Reserve	KWS veterinaries	Telephone call
February 2011	Lion	1 adult female	Maasai Mara National Reserve	KWS veterinaries	Telephone call
June 2011	Lion	3 cubs (1 male & 2 females)	Maasai Mara National Reserve (Talek)	KWS Rangers	Telephone call

Thirty-eight (68%) of the pastoralists used the following treatment or control measures when they found that their domestic animals were infected with mites: 16 (29%) sprayed or dipped their animals with acaricides, 12 (21%) reported the fact to the veterinary authorities, 12 (21%) injected teramycin, seven (13%) separated the affected individuals from the non-affected ones, four (8%) shaved the affected animals, three (5%) applied old engine oil, two (2%) injected penicillin and two (2%) injected ivermectin.

## Discussion

Our study shows that in the Masai Mara ecosystem pastoralists have knowledge of mange as a disease. As indicated by previous reports, this disease poses a health risk to both domestic animal production and wildlife conservation in this area [26]–[29]. Although pastoralists have good knowledge of the disease and are aware of its presence, the majority did not understand its aetiology as a parasite-based disease. Up to 70% of the pastoralists thought that the disease was transmitted from domestic to wild animals and vice versa. This observation has a scientific basis since cross-infections of mange between wild and domestic animals are well documented [6]. More than 85% of pastoralists had affected animals in their herds and sheep, goats, dogs and cattle were identified with mange, which agrees with previous reports of infected domestic animals in Masai Mara [27], [32]–[34].

All of the 59 wild animals reported by Masai pastoralists with visible skin lesions and signs of pruritis that were captured and whose skin was scraped were found to be positive for mange by microscopy. This confirmed that the disease reported by pastoralists was mange. Although there were no false positives, there may have been some cases of infected wild animals that were not detected by the pastoralists because it is likely that some mangy wild animal carcasses were quickly scavenged. Additionally, predators may also preferentially hunt and kill animals infected by mange, as their flight ability may be less than that of healthy animals [26]–[29]. Hence we were not able to ascertain the true mortality and morbidity rate of the affected wildlife species in Masai Mara and so it is possible that the morbidity rate was higher than the reported 59 cases.

Nevertheless, the observation that all suspected cases had mites suggests that the pastoralist reporting system is highly precise since the Masai are able to observe infected animals with skin lesions and make accurate diagnosis of mange. However, this does not reflect the sensitivity of the system. The Masai people’s knowledge of mange and their accurate identification of the disease in wild animals is an essential element in the reporting of mangy wild animals in remote areas of Masai Mara where the veterinarians and rangers of the Kenya Wildlife Service are absent. Our data highlights for the first time the importance of the Masai people in mange surveillance and reporting and so the possibility of actively including this community in disease control protocols should be fully explored.

The increased use of mobile phones may also be a way of improving the reporting of affected wild animals since they can be employed to report disease in remote areas, thereby enhancing information flow to health authorities and quick response and control.

Around two out of three pastoralists employed several treatment, prevention or control methods when they suspected the presence of mange in their herds. The majority used effective methods such as spraying or dipping their animals with acaricides. Others reported the fact to the veterinary authorities or separated the affected animals. However, the administration of antibiotics is not effective against mange and can lead to antibiotic build-up and resistance in animal tissues. Interestingly, a few pastoralists even used ivermectin, the drug of choice for mange treatment [35]–[38]. Other control methods adopted by the pastoralists such as the use of old engine oil could have arisen empirically through trial and error, although its use as a treatment or control method in mange infection in animals is not evidence-based and may be detrimental to animal health. Hence, we recommend that competent veterinary authorities provide health education to enhance pastoralists’ knowledge of disease treatment options for domestic animals. This may increase the effectiveness of the treatment given by pastoralists to their animals and facilitate collaboration with authorities in the management and control of animal diseases. The ability of Masai pastoralists to identify and treat mange can be attributed to their long experience with this disease in their livestock and with the knowledge passed down over generations. The existence of a local Masai name for mange – *olaldapash* – highlights the fact that pastoralists have been aware of the presence of this disease in their herds for many years. Furthermore, mange has been reported to occur in cheetahs, wildebeests and Thompson’s gazelles in Masai Mara [28], [29].

The fact that all of the reported mange cases in wildlife species were confirmed to be positive by the observation of clinical signs and the collection of adult mites is a demonstration of the valuable understanding of and experience with this disease that exists amongst Masai pastoralists. Additionally, the majority of pastoralists administered correct treatment to their domestic animals.

Knowledge amongst Masai pastoralists of the agents that cause mange and its treatment is usually acquired informally, being passed down from one generation to the next. One of the reasons that their livestock herds survive is that Masai fathers teach their children from an early age all the necessary skills needed for managing and protecting their herds against mange and other common diseases. Masai children usually accompany their fathers when they care for their herds and this work-shadowing is probably the most important factor in the transfer of knowledge about mange between different generations of Masai people. One of the limitations of our study is that the questionnaire did not include questions relating to the way in which mange education was acquired. Further training and active involvement in treatment and control initiatives run by government veterinary authorities should improve Masai pastoralists knowledge. Pastoralists could potentially play important roles in disease reporting and control in both wild and domestic animals.

In conclusion, we have shown that Masai pastoralists have a good understanding of both the diagnosis of mange and the necessary measures to be taken in the event of an outbreak. Our findings give a clear indication of how Masai pastoralists could be used as key informants in the early detection – as well as in the control and prevention – of mange outbreaks in both wildlife and domestic animals in Masai Mara. An integrated approach to disease control involving veterinary authorities and local pastoralists, with emphasis on the correct treatment of domestic animals and active reporting of infected wild animals, should be considered as a way of effectively controlling mange transmission in the wildlife and livestock of the Masai Mara ecosystem.

## Supporting Information

Table S1 **Questionnaire on knowledge of mange among Masai pastoralists.**
(PDF)
