# Supplementary material for: Knowledge of Mange among Masai Pastoralists in Kenya
Source: PLoS One. 2012 Aug 17;7(8):e43342. doi: 10.1371/journal.pone.0043342 (PMC3422303; doi:10.1371/journal.pone.0043342)
Supplement: Table S1 — Questionnaire on knowledge of mange among Masai pastoralists. (PDF) [file pone.0043342.s001.pdf]

Questionnaire on knowledge of mange among pastoralists

Study Area -----------------------Date…………………………………………………...

Name of Interviewer………………………………………………………………

Name of Pastoralist……………………………………………………………..

Question 1: Have you ever heard of mange?
Yes……………., No……………………

Question 2: Do you know what the etiology is?
Yes……………., No……………………

Question 3: Have you heard about mites?
Yes……………., No……………………

Question 4: (a) Have your animals been affected by this disease?
Yes……………., No……………………
(b) If yes which particular ones? ....

Question 5: (a) Are you aware if wild animals are affected by this disease?
Yes……………., No……………………
(b) If yes which particular ones? ....

Question 6: Do you think there is cross-infection between domestic and wild animals?
Yes……………., No……………………

Question 7: (a) Do you institute any control/preventive measures?
Yes……………., No……………………
(b) If yes which methods do you use? ………………………………….
